# Circular Dichroism via Extrinsic Chirality in Achiral Plasmonic Nanohole Arrays

**DOI:** 10.3390/ma19020402

**Published:** 2026-01-19

**Authors:** Francesco Floris, Margherita Angelini, Konstantins Jefimovs, Dimitrios Kazazis, Franco Marabelli

**Affiliations:** 1 Department of Physics, University of Pavia, Via Bassi 6, 27100 Pavia, Italy; francesco.floris@unipv.it (F.F.); margherita.angelini01@universitadipavia.it (M.A.); 2 Paul Scherrer Institute, 5232 Villigen, Switzerland; konstantins.jefimovs@psi.ch (K.J.); dimitrios.kazazis@psi.ch (D.K.)

**Keywords:** chirality, circular dichroism, displacement Talbot lithography, plasmonic nanohole array, Stokes parameters

## Abstract

**Highlights:**

**What are the main findings?**

- Ultrasensitive and Cost-Effective Chiroptical Platform using an achiral plasmonic nanohole array.
- Extrinsic Chirality induced by symmetry breaking under oblique light incidence.
- Scalable Fabrication using Displacement Talbot Lithography for large-area and low-cost manufacturing.

**What are the implications of the main findings?**

- Chiral Response suitable for sustaining light–biomolecule interaction for enhanced sensing.
- Promising Enantiomer Discrimination for sensitive detection in biosensing.
- First mapping of S3 for metallic structures showing interesting antisymmetric properties.

**Abstract:**

The detection of chiral properties is crucial for pharmacology and biochemistry, yet standard circular dichroism spectroscopy suffers from low sensitivity when probing minute sample volumes. While complex asymmetric chiral nanostructures can enhance these Circular Dichroic (CD) signals, their fabrication is intricate and costly. In this work, we analyzed an alternative based on extrinsic chirality in achiral square arrays of plasmonic circular NHAs realized via Displacement Talbot Lithography (DTL), thus exploring the chiroptical response arising from symmetry breaking induced by oblique illumination. Unlike isolated nanoparticles, nanohole arrays (NHAs) support propagating Surface Plasmon Polaritons (SPPs), allowing for unique light confinement capabilities essential for high-throughput sensing. A careful characterization in terms of Stokes parameters has been performed over a selected range of different optical angles of incidence and sample orientation to disentangle extrinsic chiral contribution from spurious effects related to sample imperfections. By optimizing such extrinsic chiral contributions, enhanced chiroptical response could be engineered by significantly amplifying the interaction between light and chiral biomolecules trapped within the holes. This methodology establishes DTL-fabricated achiral NHAs as an ultrasensitive, cost-effective platform for the detection and discrimination of enantiomers in biosensing applications.

## 1. Introduction

Chirality is a fundamental property describing molecules that are non-superimposable on their mirror image, i.e., enantiomers. The ability to detect and distinguish between enantiomers is of paramount importance in fields such as pharmacology and biochemistry [1,2]. The standard method for characterizing molecular chirality is circular dichroism spectroscopy, which measures the differential absorption of right and left circularly polarized light, i.e., Circular Dichroic (CD) signal.

However, conventional CD is inherently limited by low sensitivity when probing minute sample volumes or low concentrations of biomolecules—a drawback that hinders high-throughput applications and early-stage diagnostics [2]. To overcome this limitation, research has focused on amplifying the chiral signal through the interaction between the molecules and metallic plasmonic nanostructures [3]. The excitation of localized surface plasmon resonances (LSPRs) in metallic nanostructures can generate highly intensified electromagnetic fields and near-field gradients, significantly enhancing the CD signal [4].

Most approaches for CD enhancement rely on fabricating intrinsically chiral nanostructures, characterized by an asymmetric 3D geometric shape. While these structures (such as helices, spirals, or asymmetrically arranged metastructures) have shown remarkable enhancement factors, their fabrication is often complex, costly, and not scalable, typically requiring advanced techniques like Electron Beam Lithography (EBL) or multi-step self-assembly [5]. This complexity limits their widespread adoption for cost-effective sensing platforms.

A robust alternative is offered by the concept of extrinsic chirality. A geometrically achiral structure (one that possesses a mirror-symmetry plane) can exhibit chiroptical activity (and thus CD) when illuminated in a manner that breaks the overall symmetry. This occurs, for example, under oblique illumination (non-normal incidence) on planar metastructures, where the entire system (structure + light propagation direction) becomes chiral [6,7,8]. Extrinsic chirality offers the advantage of utilizing simpler, mass-producible nanostructure geometries.

In this work, we present a promising alternative for cost-effective sensing platform based on extrinsic chirality in achiral square nanohole arrays (NHAs) of circular plasmonic nanoholes able to support the propagation of Surface Plasmon Polaritons (SPPs) [9]. SPP resonance is highly sensitive to refractive index changes and enables unique light confinement capabilities, which are essential for maximizing the light–biomolecule interaction in high-throughput sensing applications.

Crucially, the fabrication of these arrays is achieved via Displacement Talbot Lithography (DTL) [10], which is a large-area, parallel, and low-cost patterning technique that offers superior scalability and speed compared to EBL or Focused Ion Beam (FIB) methods.

We demonstrate that by breaking the system symmetry through oblique illumination, it is possible to engineer enhanced chiroptical response within the nanoholes, which act as optical traps and sensing chambers for biomolecules. Through the optimization of the array periodicity and hole diameter, a resonant coupling between the SPP resonance and the extrinsic chirality can be achieved. Our results establish DTL-fabricated achiral NHAs as a potential innovative platform for the detection and discrimination of enantiomers in biosensing applications [11], providing a scalable alternative to complex intrinsic chiral nanostructures.

## 2. Materials and Methods

### 2.1. Displacement Talbot Lithography

DTL is a high-resolution photolithography technique that combines the benefits of compact, mask-based Talbot lithography with the high depth of focus of interference lithography, enabling robust nano-patterning on a wafer scale. DTL specifically addresses the stringent flatness requirements typical of proximity lithography by continuously changing the distance between the mask and the wafer during exposure, as demonstrated by Solak et al. [12]. Such a process averages the intensity along the Talbot pattern propagation, rendering the resulting pattern independent of the specific mask-to-wafer distance. This ensures a uniform dose across the substrate, significantly enhancing the technique’s robustness and scalability for large-area production [13].

Considering this, we employed an achiral NHA like that formerly studied by Angelini et al. [14]. Our aim, extending the analysis conducted by Pellacani et al. [15], was to subject circular holes arranged in a square array, i.e., the highest symmetric scatterer shape and array disposition combination, to oblique incidence conditions, specifically targeting the verification of any resultant CD signal arising from extrinsic chirality. In our case, we aimed at patterning the square NHA within a gold film with a thickness of 80 nm, a hole diameter of 190 nm, and a target pitch (P_target_) of 450 nm. The aerial image produced by DTL corresponds to a square hole array rotated by 45° with respect to the mask pattern, with the pattern pitch reduced by a factor of $1/\sqrt{2}$ relative to the mask pitch (P_mask_). Consequently, we used a mask with P_mask_ = 635 nm, satisfying the relationship $P_{mask}=\sqrt{2}\;P_{target}$.

The fabrication process for this NHA is identical to that described in [15]; for convenience, the details are provided in Appendix A along with a SEM image of the investigated sample.

### 2.2. Optical Characterization

**Figure 1:**
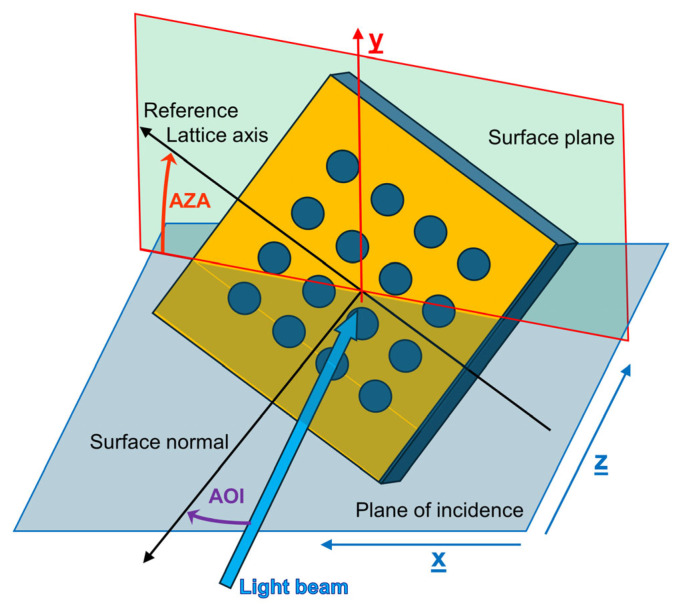
Sketch of the sample optical orientation reporting the main axis and planes.

To study the extrinsic chiral response of the NHA under investigation, angle-resolved reflectance (R) and transmittance (T) were measured for different angles of incidence (AOIs) and several azimuthal angles (AZAs). Their geometrical definitions are shown in Figure 1, together with the main axis and planes.

All the measurements were performed resorting to a Bruker (Billerica, MA, USA) IFS66s Fourier Transform interferometer coupled to a custom-built reflectometer. For convenience and consistency, when evaluating the CD signals, both the R and T signals were collected through a single non-polarizing beam-splitter cube and directed to a Si photodetector as reported by Angelini et al. [16]. This setup allowed for illuminating the sample surface with a focused beam, characterized by a spot size diameter of 200 μm and a divergence angle lower than 1°. To collect the Stokes polarization parameters, the setup was, instead, modified to adhere to the so-called rotating quarter-waveplate method, reported by Schaefer al. [17]. In fact, such a method is particularly advantageous thanks to its accuracy and efficiency, allowing a simple curve fitting algorithm to be used to determine the Stokes parameter, thus avoiding both complexity and limitations of the classical method. The details of the optical setup and its schematics are reported in Appendix B.

Diffuse light measurements were performed using an Agilent Technology (Santa Clara, CA, USA) Cary 6000i spectrophotometer at normal incidence resorting to its built-in Agilent Technology (Santa Clara, CA, USA) Ulbricht integrating sphere tool.

### 2.3. Optical Analysis

According to the literature, CD was defined as

(1) $$CD=\frac{A_{left}-A_{right}}{A_{left}+A_{right}}$$

where A_left_ and A_right_ indicate the absorbance for left- and right-handed circularly polarized light, respectively. Thanks to the quality of the array, the low divergence of the beam and the absence of diffracted beams in the spectral region under investigation, A signals were evaluated as

(2) $$A_{left,right}=1-R_{left,right}-T_{left,right}$$

Furthermore, the accurate determination of Stokes parameters requires precise calibration of the polarimetric system, which is subject to uncertainties stemming from the alignment of components and the actual phase retardation value of the wave plates.

In fact, the misalignment of the Quarter-Wave Plate (QWP) by an angle Δθ relative to the reference frame causes the λ/4 phase retardation to be incorrectly applied to rotated electric field components, which translates into significant crosstalk among the Stokes parameters, mixing polarization components (e.g., causing circular polarization to appear as linear). Additionally, the misalignment Δγ of the linear polarizers (in both the state generator and the state analyzer) further exacerbates the issue, introducing additional systemic error terms and crosstalk into the Stokes parameters, which is essential for correlating the measured intensities to the true Stokes vector.

Finally, a critical source of error is the inherent uncertainty in the actual phase retardation value δ of the QWP, which may deviate from the nominal π/2 radians (a λ/4 shift) due to manufacturing or environmental factors. Thus, a rigorous calibration procedure, as reported by Marcías-Romero and Török [18], was applied to both alignment errors (Δθ, Δγ) and retardation errors (Δδ) to achieve accurate polarization measurements.

## 3. Results and Discussion

**Figure 2:**
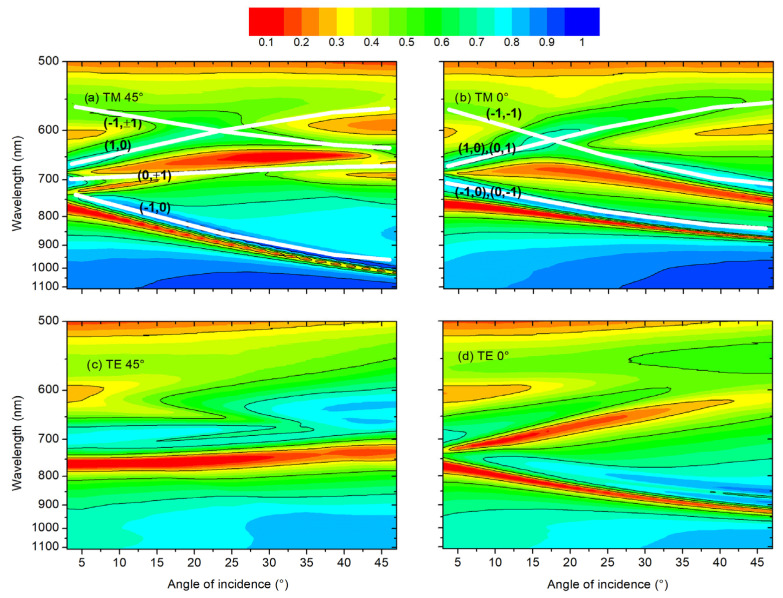
Dispersion of the plasmonic modes as measured by reflectance with polarized light parallel (TM) or perpendicular (TE) to the plane of incidence when aligned along the square lattice directions at 0° or its diagonal at 45°. For convenience, the SPP modes have been labeled, and their dispersion is indicated by white lines.

Our initial analysis started from the TE and TM maps for R in the 3–47° AOI range to figure out the plasmonic mode dispersion. Figure 2 illustrates the development of plasmonic modes along the principal lines of symmetry (crystal axes) and the diagonal.

The R spectra in Figure 2 are primarily dominated by the dispersion of SPP modes. However, these samples exhibit strong interaction and hybridization between SPP and Localized Surface Plasmon Resonance (LSPR) modes. This coupling is particularly evident near normal incidence around 700 nm, where the convergence of various plasmonic branches towards normal incidence occurs and, additionally, the lifting of degeneracy in pure SPP modes leads to the formation of a plasmonic bandgap [19].

### 3.1. Chiroptical Response and Dichroism Effects

**Figure 3:**
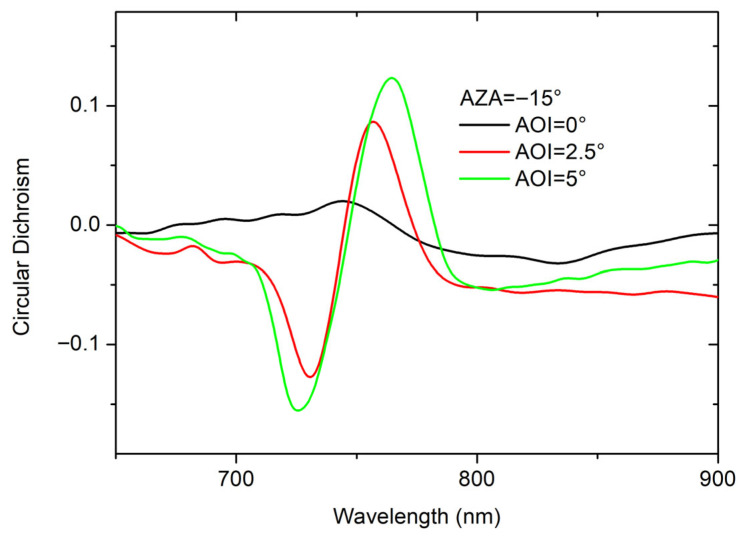
Circular dichroism measured at AZA = −15° for AOI = 0°, 2.5°, and 5°.

Given the characteristics identified in the band structure, attention was focused on the behavior of the system for AOI close to 0°, where the chiroptical effects, specifically CD, are predominantly localized within the spectral region defined by the photonic bandgap. To investigate this, we analyzed the CD response at three key incidence angles: AOI = 0°, AOI = 2.5°, and AOI = 5°. As the AOI increases, the CD signal becomes significantly more structured, as shown in Figure 3.

**Figure 4:**
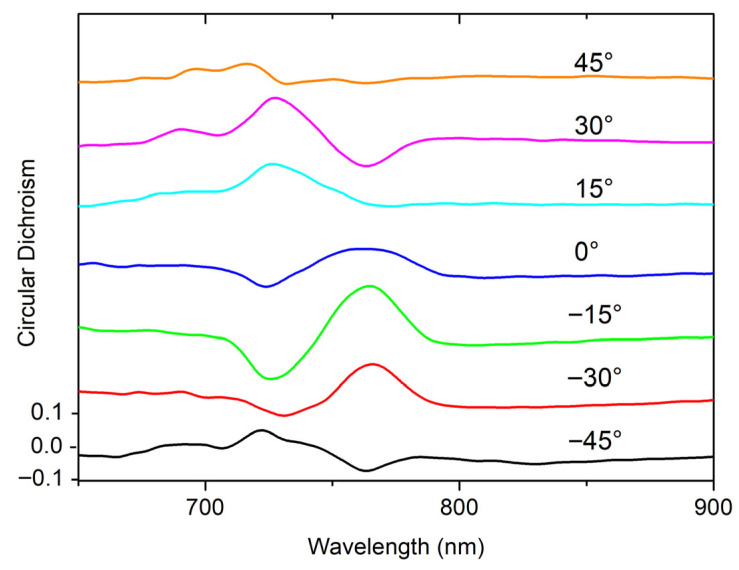
Circular dichroism measured at AOI of 5° for different AZAs. AZA = 0° refers to the sample orientation along the square lattice direction.

Meanwhile, the CD signal progressively exhibits a strong dependence on the AZA as shown in Figure 4 for AOI = 5°. The correspondent data for AOI = 2.5° are reported in Appendix C.

While the absolute value of the measured CD is not exceptionally high, it can be maximized by rotating the AZA, reaching absolute values up to 0.2. This strong AZA dependence is a particularly interesting effect that facilitates polarization tunability via simple mechanical rotation and is characteristic of extrinsic chirality induced by the oblique incidence, where the in-plane symmetry is explicitly broken by the experimental geometry.

Crucially, a small, yet detectable, CD signal is observed even at AOI = 0° (i.e., normal incidence) and AZA = 0° or ±45° (i.e., aligned along the lattice symmetry directions). Theoretically, for these conditions, structures possessing in-plane symmetry should exhibit zero CD due to symmetry constraints and, therefore, the observation of this small non-zero signal can be ascribed to a residual misalignment or either a minor fabrication imperfection or sample tilt that breaks the in-plane symmetry.

### 3.2. Correction for Scattered Light

**Figure 5:**
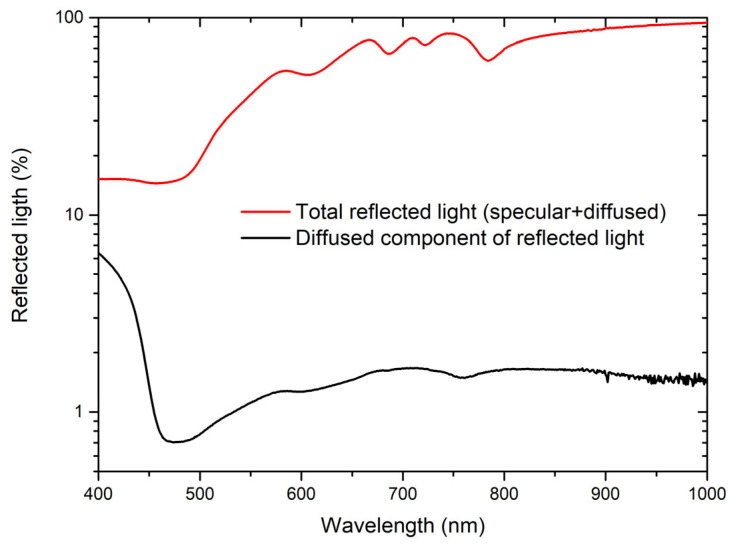
Total reflected (red curve) and diffuse (black curve) light intensities collected using an integrating sphere.

The observed chiroptical effect does not consider the influence of scattered light, not accounted for in Equation (1). To verify such an effect, arising from lattice diffraction or, in general, from surface roughness, we estimated the amount of diffused light, as illustrated in Figure 5. A steep increase in the diffuse component can be observed, ascribable to diffraction, with respect to the total reflected light for wavelengths smaller than 450 nm, which is consistent with the P_target_ value. In the spectral region of interest between 500 and 1000 nm, a diffused component is measured with values between 1 and 2%, roughly proportional to the total reflectance. Such an effect does not affect the behavior of DC signal while it influences its absolute value, and the subtraction of a background due to diffused light tends to increase the CD values; then, our CD is underestimated.

### 3.3. Stokes Parameters Analysis

An alternative approach to study the chiral effect implies the determination of the Stokes parameters (SPs) [20], particularly S3 [21], that can be performed with transmittance measurements, implicitly considering also spurious effects arising from depolarization or diffusion.

**Figure 6:**
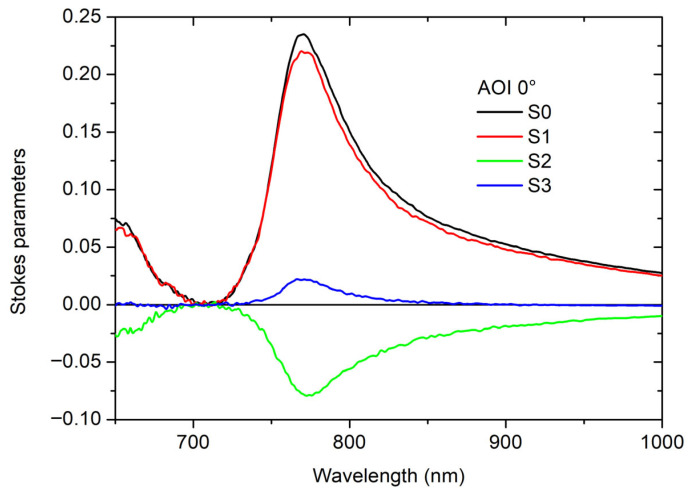
Non-normalized Stokes parameters as obtained at AOI = 0° and AZA = 45°.

In this way, the complex dependence on AOI and AZA can be further investigated. The full set of SP spectra measured at AOI = 0° and AZA = 45° values is presented in Figure 6.

The analysis of SPs provides a comprehensive quantification of the polarization state, allowing us to map the transitions between linear, elliptical, and circular polarization components as a function of wavelength, also complementing the CD measurements by revealing the nature of the induced ellipticity and rotation across the bandgap region.

For effective comparison, normalized SPs are typically required to be the normalized S3 directly associated with CD, i.e., CD = S3 as reported in [21,22].

However, we present the not-normalized values (as referenced in Figure 6) because the total intensity (S0) approaches zero within the photonic bandgap and normalizing the parameters (S1/S0, S2/S0, S3/S0), in this regime, would cause the curves to diverge significantly due to noise fluctuations, severely obscuring the underlying physical phenomena and making the visualization of the data less effective; the not-normalized maps, therefore, provide a more robust representation of the polarization state variations in the vicinity of the bandgap.

**Figure 7:**
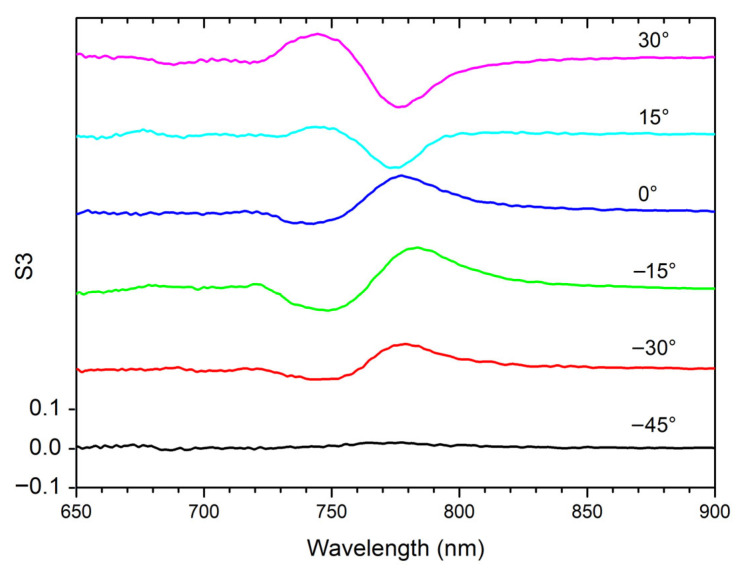
Not-normalized S3 spectra collected at AOI = 5° for different AZAs.

The S3 spectra, collected at AOI = 5° for different AZA values, are presented in Figure 7.

It can be interesting to compare such S3 spectral behavior with the CD curves (Figure 4), showing a good agreement in terms of evolution with the AZA.

**Figure 8:**
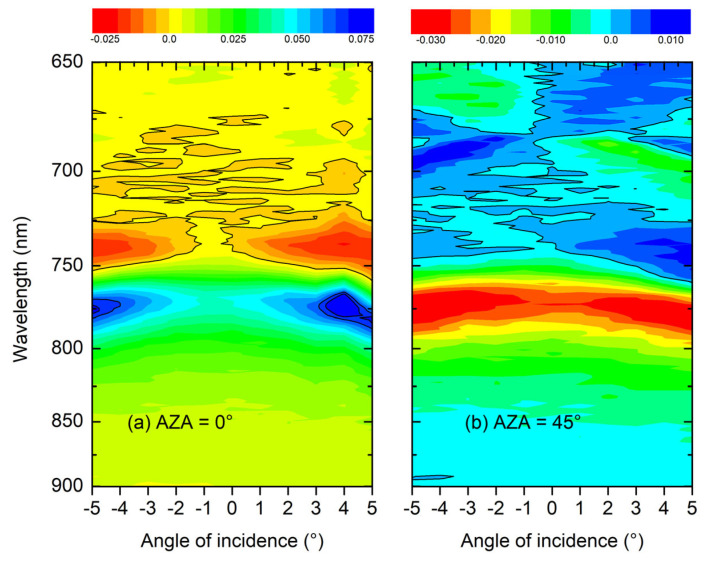
Chiral maps showing the not-normalized S3 at AZA = 0° (**a**) and AZA = 45° (**b**).

An effective way to summarize chirality as a function of AOI is obtained by plotting the maps of S3 vs. wavelength; see Figure 8.

The point of symmetry, the real zero for AOI, is much better identified, particularly in the case where the AZA is 0°, where the normal incidence, in this specific configuration, is shifted by roughly 1°, and a similar observation holds for AZA = 45°. The dispersion is revisited near the AOI = 0°, which connects back to the observations presented in Figure 2.

In this specific context, we can estimate the values between AOI = 0° and AOI = 5°. A play of symmetries is observed when AZA is 0°; a symmetry is visible for positive and negative AOI while, conversely, for AZA = 45°, at wavelengths lower than 750 nm, an antisymmetric behavior of the signal is observed for positive and negative AOI.

It is worth highlighting that for a more detailed analysis of these symmetry-breaking effects, it would be necessary to perform an accurate photonic band calculation for our specific metal–dielectric structure, as demonstrated for purely dielectric systems by Zagaglia et al. [23]. While their methodology provides a robust framework, the inclusion of metallic components in our structure introduces significant differences, notably the presence of SPPs and the associated losses, which drastically influence the band structure and dispersion features. A dedicated calculation would be crucial to accurately map the resonant modes and precisely define the observed zero points and dispersion trends; while currently out of the scope of this specific work, performing this simulation is planned for a future study.

**Figure 9:**
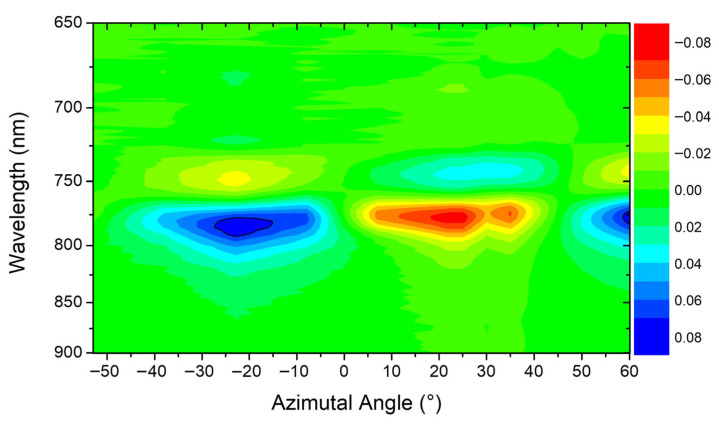
Chiral map showing the not-normalized S3 at AOI = 5°.

The discussion becomes even more precise when the results are reconsidered as a function of the variation in the AZA, as proposed in Figure 9.

The results comprise two distinct datasets acquired during separate experimental sessions. The first is a broad-range set, which illustrates the general signal evolution with a 15° resolution. The second is a high-resolution set, which focuses on the symmetry point (30–60°) with a finer 5° resolution. To correct for a slight experimental offset observed in the 15° resolution data relative to the theoretical symmetry angles, we centered the AZA = 0° position at the minimum signal region (the transition between positive and negative values). This combined visualization reveals different S3 maxima and minima occurring between ±15° and ±30°. This extremes values align with the ~±20° peak observed in the map and corresponds to the maximum displacement from symmetry conditions, consistent with the previous literature [23,24].

This AZA acts as a critical parameter controlling the in-plane symmetry of the excitation. It is evident that as one moves away from the primary axes of symmetry (i.e., AZA = 0°, 90° or AZA = ±45°), the underlying structural symmetry is broken in the context of the incident light wavevector. It can also be noted that the map representation allows for better identification of the symmetry line, identifying the AZA as a powerful tuning knob for controlling the handedness and magnitude of the CD in such metastructures.

Lastly, it is fundamental to properly consider the impact of a correct alignment of the array with respect to the polarization state of the incident light. In fact, the small CD measured at AOI = 0°, as discussed in Section 3.1, could be related to tight alignment precision.

**Figure 10:**
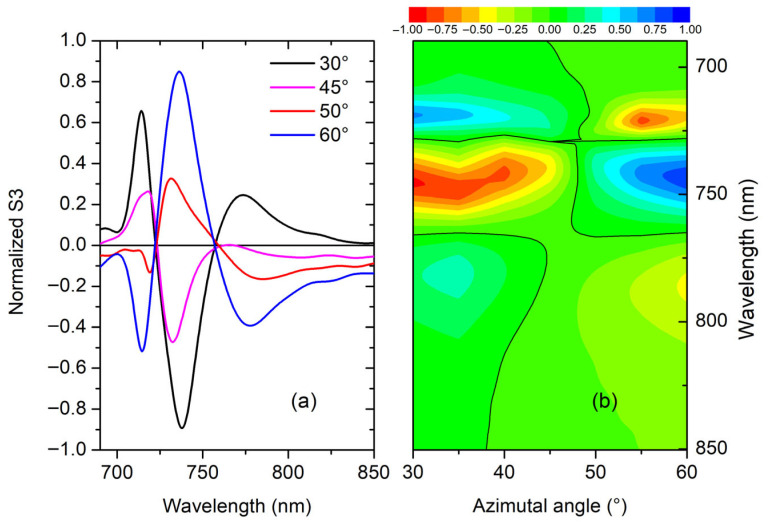
Normalized S3 for AOI = 5° plotted as curves, panel (**a**); chiral map, panel (**b**), in the AZA range from 30° to 60°, focused on the wavelength interval comprised between 690 nm and 850 nm, including the structures showing the most marked CD response.

Moreover, when considering the chiroptical effect through the normalized S3, represented in Figure 10, the obtained values become close to unity reinforcing the potential application to sensing.

**Figure A4:**
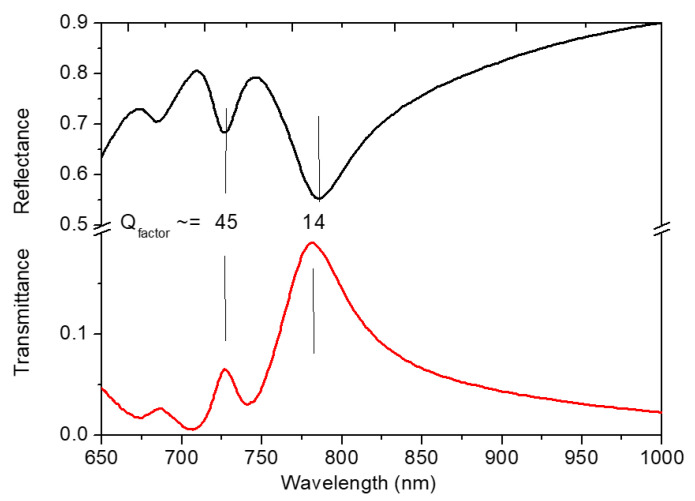
Quality Factor evaluated for the two resonances involved in the chiral response at 780 and 730 nm.

In this respect, the experimental Quality Factor (Q_factor_) serves as a primary parameter for evaluating performance. Based on the T and R line shapes, the Q_factor_ for the two resonances involved in the chiral response at 780 nm and 730 nm are estimated to be 14 and 45, respectively, as shown in Figure A4 in Appendix D. Notably, these values compare favorably with those reported in previous studies [25].

Since the Q_factor_ is intrinsically linked to field enhancement, which is generally estimated to be in the order of 10 for similar NHAs [26], our relatively larger Q_factor_ values suggest the potential for equal or superior enhancement levels.

While high field enhancement is a prerequisite for sensitive chiral molecule detection, performance must be evaluated comprehensively. The literature suggests that chiral sensitivity is governed by two key features: (i) the molecule’s inherent chirality (coupling between electric and magnetic dipoles), and (ii) the chirality of the electromagnetic field, which can be engineered via surface structures [27,28].

Consequently, the chirality induced by the surface itself is a critical metric.

A structure like the one studied here was recently investigated for enantiomer discrimination [29]. Numerical simulations in that study demonstrated that an AOI of 10° and an AZA of 30° break field symmetry, leading to a chiral enhancement factor of approximately 20 and, notably, the spectral behavior of this enhancement closely matches the CD features.

Given the similar geometries of our structures to those in [29] (with differences primarily in fabrication method and hole size), we applied the same criteria to our samples. To facilitate direct comparison, we calculated the Chiral Transmission Dichroism (CD_T_) using the following definition:

(3) $$CD_{T}=\tan^{-1}\left(\frac{T_{right}-T_{left}}{T_{right}+T_{left}}\right)$$

**Figure A5:**
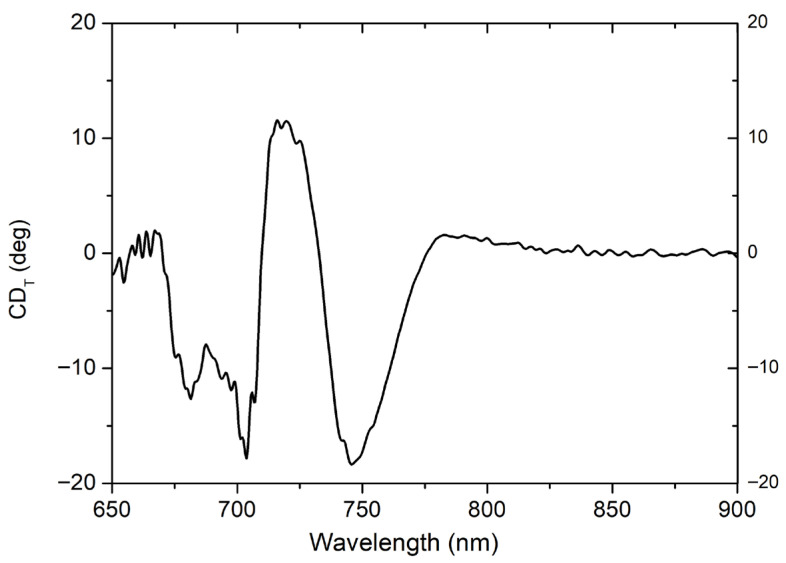
Sensing performance evaluated as chiral transmission dichroism applying Equation 3.

The resulting signal obtained in our case is one order of magnitude larger than the comparative study, as can be seen in Figure A5 reported in Appendix D, and we are therefore confident that the sensitivity tests planned soon will provide compelling results for chiral molecule detection.

## 4. Conclusions

This work establishes the efficacy of achiral NHAs fabricated via DTL as a scalable, cost-effective platform for extrinsic chirality. We demonstrated that symmetry breaking induced by oblique illumination generates a robust chiroptical response, with CD strongly dependent on the AOI and AZA values. The analysis of Stokes parameters, particularly S3, provided a precise quantification of the response, revealing antisymmetric properties, and achieving normalized values close to unity. This angular tunability and high induced efficiency envision DTL-NHAs as a scalable solution for chiroptical properties engineering. In summary, the methodology offers a promising platform for enantiomer discrimination and biosensing applications, and we are confident that sensitivity tests, planned soon, could provide interesting results for chiral molecule detection.

## Figures and Tables

**Figure A1:**
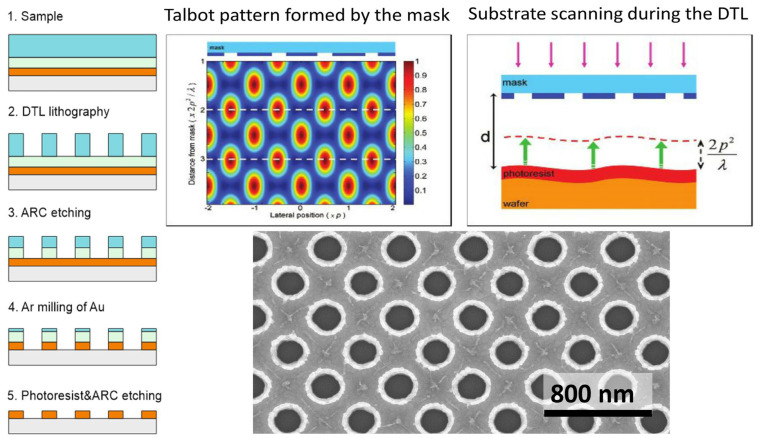
Left—Schematics of the fabrication process; Top Center—Diffraction of the beam on the periodic pattern known as the Talbot carpet under the mask; Top Right—Substrate scanning to ensure a uniform dose in the nanoholes; Bottom Right—SEM image of the measured square array with Au thickness 100 nm, pitch 450 nm, and hole diameter 200 nm.

**Figure A2:**
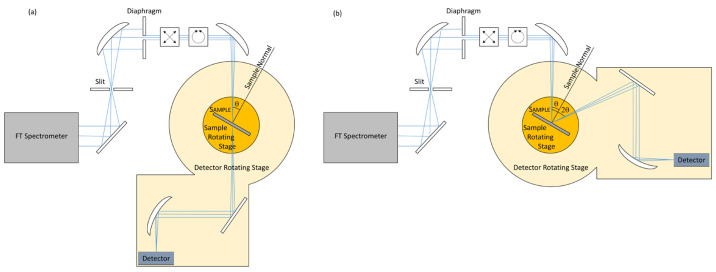
Schematics of the optical configuration. Transmission and specular-reflection schemes are depicted in panel (**a**) and panel (**b**), respectively.

**Figure A3:**
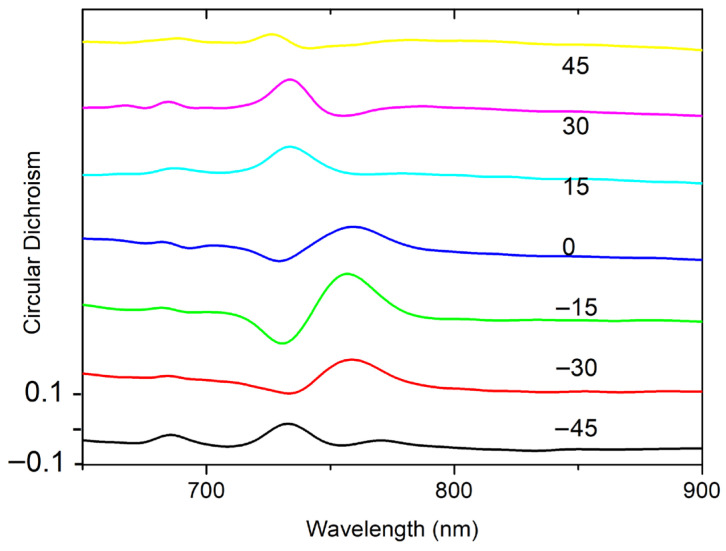
Circular dichroism measured at AOI = 2.5° for different AZAs. AZA = 0° refers to the sample orientation along the square lattice direction.

## Data Availability

The original contributions presented in the study are included in the article. Further inquiries can be directed to the corresponding author.

## Abbreviations

The following abbreviations are used in this manuscript:

NHA	NanoHole Array
DTL	Displacement Talbot Lithography
CD	Circular Dichroism
SPs	Stokes Parameters
AOI	Angle Of Incidence
AZA	AZimuthal Angle
R	Reflectance
T	Transmittance
A	Absorbance

## Appendix A. Displacement Talbot Lithography

Substrates were 100 mm diameter, 500 μm thick round Schott glass wafers. A 5 nm layer of Cr (used as an adhesion layer) and a 100 nm layer of Au were deposited onto the wafers by electron beam evaporation (Evatek BAK Uni, Aschheim Dornach, Germany). The wafers were then coated with an AZ BARLI-II antireflective layer (MicroChemicals GmbH, Ulm, Germany) and a PFI-88A7 photoresist layer from SUMIRESIST (Figure A1—Left, Step 1). The DTL exposures were performed using a PhableR 200C tool (Eulitha AG, Würenlos, Switzerland) with 377 nm wavelength and unpolarized light.

The diffraction of the beam on the periodic pattern forms the so-called Talbot carpet under the mask (Figure A1—Top Center). In classical Talbot lithography, the sample is placed in one of the Talbot self-image planes to obtain the exposures, which is very demanding over large areas due to very narrow mask-to-sample distance tolerances. This problem is resolved in the displacement Talbot regime, where the sample is continuously moving along the beam propagation direction.

The Talbot distance, calculated using the equation d = 2P^2^/λ, was approximately 2.14 μm. During the 60 s exposure, the substrate was scanned over 10 Talbot distances (about 21.4 μm) to ensure a uniform dose in the nanoholes (Figure A1—Top Right). After exposure, the PFI-88A7 photoresist was developed in MF24A (Figure A1—Left, Step 2), and the BARLI-II layer was etched using reactive ion etching in an oxygen plasma (Figure A1—Left, Step 3). The Au layer was then etched by Ar ion milling in an Oxford IonFab300 tool at a 10° angle on a rotating chuck (20 rpm), with a beam current of 55 mA and voltage of 300 V for 12 min (Figure A1—Left, Step 4). The remaining organic layers (BARLI-II/PFI-88A7) were removed by oxygen plasma (Figure A1—Left, Step 5). A final thermal annealing was performed keeping in an oven the sample for 60 min in air at 300 °C to make uniform and compact the gold layer and smooth the surface by removing some residual irregularities induced by Ar ion milling in the proximity of nanoholes edges. The resulting structures were analyzed resorting to SEM images (Figure A1—Bottom Right) to verify that the surfaces created adhere to the parameters identified during the optimization phase.

## Appendix B. Optical Setup Scheme

The scheme of the optical setup is depicted in Figure A2, panel (a) reporting the T configuration and panel (b) showing the specular-R one. The electromagnetic radiation exiting the FT spectrometer is collected by a mirror and focused onto a slit to adjust its size to the desired one. Then, a diaphragm selects the source beam aperture while a polarizer and a QWP set the proper polarization state to be focused onto the sample, which is placed onto an XYZ translator and a rotation stage at the same time. The rotation stage allows the variation in the AOI of the light beam while a homemade sample holder enables the rotation of the sample with respect to the surface normal selecting the AZA. The sample surface is visualized by a camera, enabling the correct positioning of the sample on the axis of rotation, and selecting the surface area to investigate. The reflected or transmitted beam is then collected by a Si-detector.
